# Controlling compartmentalization by non-membrane-bound organelles

**DOI:** 10.1098/rstb.2017.0193

**Published:** 2018-04-09

**Authors:** Richard J. Wheeler, Anthony A. Hyman

**Affiliations:** Max Planck Institute of Molecular Cell Biology and Genetics, Pfotenhauerstraße 108, Dresden, Germany

**Keywords:** liquid–liquid phase separation, biomolecular condensates, non-membrane-bound organelle, cell compartmentalization

## Abstract

Compartmentalization is a characterizing feature of complexity in cells, used to organize their biochemistry. Membrane-bound organelles are most widely known, but non-membrane-bound liquid organelles also exist. These have recently been shown to form by phase separation of specific types of proteins known as scaffolds. This forms two phases: a condensate that is enriched in scaffold protein separated by a phase boundary from the cytoplasm or nucleoplasm with a low concentration of the scaffold protein. Phase separation is well known for synthetic polymers, but also appears important in cells. Here, we review the properties of proteins important for forming these non-membrane-bound organelles, focusing on the energetically favourable interactions that drive condensation. On this basis we make qualitative predictions about how cells may control compartmentalization by condensates; the partition of specific molecules to a condensate; the control of condensation and dissolution of condensates; and the regulation of condensate nucleation. There are emerging data supporting many of these predictions, although future results may prove incorrect. It appears that many molecules may have the ability to modulate condensate formation, making condensates a potential target for future therapeutics. The emerging properties of condensates are fundamentally unlike the properties of membrane-bound organelles. They have the capacity to rapidly integrate cellular events and act as a new class of sensors for internal and external environments.

This article is part of the theme issue ‘Self-organization in cell biology’.

## 1. Introduction

In order to organize their biochemistry, cells form compartments. Many are bound by membranes, and these tend to be stable. Composition of these compartments is defined by membrane transporters and porins (table 1). These proteins control which small molecules, proteins and other biological polymers can access the compartment. However, many cellular compartments are not bound by membranes, and these tend to assemble and disassemble rapidly. They can form in the cytoplasm or nucleoplasm, but here, for simplicity, we generally refer to the cytoplasm. These compartments can have solid, gel or liquid-like properties. Many are liquid-like, and form through phase separation [1–6]. This arises from the polymer nature of proteins [7,8].

**Table 1.** The differences between the dynamics and composition control of membrane-bound and liquid non-membrane-bound organelles.

organelle type	formation	destruction	composition	merging
membrane-bound organelle (mitochondria, endoplasmic reticulum, lysosome, etc.)	membrane budding (fission from other compartments), vesicle fusion	autophagy, fusion with other compartments, fission to vesicles	passive/active transporters, porins	membrane fusion
pseudo-membrane-bound organelle (nucleus)	topology change from single membrane-bound organelle, vesicle fusion	topology change, fission to vesicles	membrane openings (nuclear pore)	fusion with membrane joining
condensate organelle	condensation from solution, control of nucleation	dissolution into solution	solute partition	miscibility

A homogeneous mixture of polymer and solvent can separate by condensation to form a polymer-enriched phase that coexists with a polymer-depleted solution. This is well characterized for synthetic polymers, especially homopolymers or block-copolymers. The physics of phase separation is proving to be relevant and important for biological polymers made of a number of different monomers. In some ways, formation of these compartments is similar to condensation of water droplets from water vapour. In other ways, the droplets formed are similar to a droplet of oil that is immiscible with surrounding water. However, neither are precise analogues. We refer to this class of non-membrane-bound compartments as *biomolecular condensates*, or simply *condensates* for convenience [7]. To a cell, these condensates may seem solid or liquid. This depends on viscosity and viscoelasticity, as very high viscosity liquids with elastic recoil may have solid-like behaviour on the time scales of cellular dynamics (seconds to minutes). It also depends on whether the polymer becomes kinetically trapped in a gel or glass-like state with more solid properties. Here we focus on liquid states.

For a cell to exploit condensates as biochemical compartments, they must: (i) control when they form, and when they dissolve (disassemble); (ii) control their composition, and control what should be excluded. In this review, we discuss how our current knowledge of the protein interactions driving condensate formation allows us to begin to predict how a cell may exploit a condensate as a biochemical compartment. Many of these predictions are built on incomplete evidence, or a greatly simplified view of the complex environment of a living cell that is decidedly out of equilibrium, but they provide an equilibrium touchstone for framing a discussion about non-equilibrium effects.

## 2. Proteins can form condensates

Most generic synthetic polymers undergo phase separation in aqueous solution. This is also true of biological macromolecules in cells, with many examples for proteins [1–6] and one example for nucleic acids [9,10]. The physical chemistry driving this behaviour has previously been reviewed in detail [11]. The core concepts are that the highest entropy state is a well-mixed solution of polymer in solvent. Phase separation, to make condensates, generates a lower entropy state. Therefore, some energetic benefit of intermolecular interactions must exist to overcome this entropic cost. This energetic benefit comes from the balance of three interactions: solvent–solvent, solvent–polymer and polymer–polymer. Energetic benefits from favourable solvent–solvent and polymer–polymer interaction can favour phase separation. The interactions driving phase separation must be sufficiently energetically favourable to overcome the entropic cost of phase separation. Multiple sites on a polymer where favourable polymer–polymer interactions can occur can support such a scenario. Interactions cannot be so strong as to become permanent on time scales relevant to cell dynamics (minutes or seconds), which would make a solid phase. Using typical biochemical terminology, suitable polymer–polymer interactions to drive phase separation are multivalent, strong and yet ‘transient’ on timescales that are relevant for biochemical reactions and cellular processes.

The ability to phase separate is not unusual for a polymer and it is likely many proteins can be made to phase separate under some conditions. Therefore, to ensure biological relevance of a condensate, its formation must occur under physiological conditions. We suggest low μM polymer (protein) concentration [12], moderate salt concentration (around 100 mM KCl) and a physiological pH (pH 7.4). The cytoplasm is a crowded environment owing to high protein concentration. This can contribute to phase separation by a variety of modes, including so-called depletion-mediated effective attractions whereby the proteins are forced together in cohesive interactions because the crowding molecules deplete the free volume available to the proteins in solution. Therefore, depletion-mediated attractions refer to an effective binding energy, arising from the exclusion of the crowding molecules between two polymers [13]. Additionally, crowding molecules can also have a symmetry-breaking effect whereby they impact the physical dimensions of individual proteins, which in turn enables interactions that would be unavailable in dilute solutions [14]. Low concentrations (less than 5%) of a crowding agent (dextran, polyethylene glycol, glycogen) can be included. However, high concentrations are not physiological. Just 2% glycogen provides sufficient crowding for normal spindle aster formation in *Xenopus* egg extract [15]. Ultimately, for confidence, direct biological evidence is needed, such as the protein being an abundant component of a condensate in cells.

## 3. Classes of condensate-forming proteins

There are two main classes of protein that form condensates under physiological conditions [7]: Proteins with intrinsically disordered regions (IDRs) that also include *low complexity domains (LCDs)*, and proteins made up of *multiple copies of interaction domains (MCIDs)*. Not all IDRs are LCDs, and given that many LCDs also form rod-like alpha helical structures, it also follows that not all LCDs are IDRs. Further, the designation of an LCD minimizes hidden complexities that are intrinsic to many sequences, but we use this term here to aid in familiarity and remain consistent with a trend that has emerged in the literature. There are several well-characterized examples of condensate-forming proteins with so-called LCDs; the stress granule proteins FUS, hnRPA1 and TDP43 [3,16–18], which are RNA binding proteins that require the LCD for condensate formation; and the Nephrin intracellular domain [19], which is a long IDR that is part of a transmembrane signalling protein. This region may or may not be well described as an LCD. There are also two well-characterized examples of condensate-forming proteins with MCIDs; the Nephrin/Nck/N-WASP signalling complex and the LAT/Grb2/Sos1 signalling complex. Both depend on specific heterotypic interactions of SH2 domains with phosphotyrosine residues and SH3 domains with proline-rich sequences [4,5].

These two classes, IDRs/LCDs and MCIDs, are superficially very dissimilar. However, both appear to drive energetically favourable multivalent protein–protein interactions that allow phase separation to make condensates (see below). These proteins have been termed *scaffolds*. ‘Scaffold’ does not refer to a rigid structure; instead it refers to the role of the protein as a hub for multivalent interactions that are required for phase separation. A protein or group of proteins necessary for phase separation to form a condensate can be viewed as scaffold(s). A scaffold protein may have both MCIDs and LCDs, which may both contribute to phase separation.

The leading model for condensate formation by LCD-containing proteins is many energetically favourable cross interactions between amino acids [11]. An LCD has low sequence complexity, and this can confer a low propensity for stable secondary or tertiary structure, and more uniform chemical properties from a low diversity of amino acids. This gives a high local concentration of amino acid side chains with particular properties, and enables multivalent interactions. It may also expose residue types that are normally buried within a protein tertiary structure. For example FUS phase separation requires tyrosine residues (hydrophobic and aromatic) in the FUS LCD [20]. Phase separation of the Nephrin intracellular domain requires many complementary charged (acidic and basic) residues [19].

LCDs tend to fall into classes with characteristic amino acid properties, notably polar tracts, polyampholytes or polyelectrolytes [21]. Significant effort has been put into identifying how amino acid properties in a LCD contribute to condensate formation [21–24]. Energetically favourable interactions between amino acid residues likely drive phase separation. Unfortunately, few have been comprehensively analysed experimentally. However, the dominant interactions are likely to be positive/negative charge, hydrophobic, positive-pi orbital [25], and pipi stacking [11]. Characteristic strengths and distance scales for these interactions are on the order of 0.1–1 kJ mol^−1^ and 0.1–1 nm (the size of amino acids), but any interaction is likely strongly influenced by local environment.

The diversity of amino acid properties complicates theoretical prediction of phase separation of LCD-containing proteins. Some theoretical understanding may be drawn from the field of protein folding. For example, for a condensate formed owing to favourable hydrophobic interaction there may be parallels with the molten globule state during protein folding. This is a disordered dynamic and liquid-like state with hydrophobic sections of the peptide tending to self-interact, shielded from the surrounding water solvent by hydrophilic sections [26]. Predictions must also incorporate the ability of some residues to undergo favourable homotypic interaction, while others require heterotypic interaction. In particular, for charge–charge interactions an individual residue cannot self-interact (as like charges repel). However, a basic (positive) protein and one acidic (negative) protein can strongly interact. In this case, the condensate made up of the positive and negative charged polymers is termed a coacervate [19]. This class of condensates likely includes nucleolar and heterochromatin compartments, which both have liquid properties [1,27–30]. In these cases, the positive charged polymers are proteins and the negative polymers are likely both nucleic acids and negative charged proteins.

A second possible mechanism for condensate formation by LCD-containing proteins is spontaneous fluctuations into and out of secondary structural elements, occurring with some probability [16]. Particular LCDs tend to form particular secondary structures when ‘solidified’ in a tertiary structure. Prion-like LCD amino acid composition [31,32] tends to have beta sheet-rich structures [33]. There is mixed evidence for the presence of secondary structure elements in liquid condensates, including alpha helical structure for the TDP43 LCD [16] and stacked beta sheets for FUS and hnRNPA2 LCDs [20,34], although other evidence suggests a completely disordered state [35]. It is possible that secondary structures might contribute to kinetically stable gel or glass-like compartments or solid fibres under other cellular conditions or on longer time scales.

Proteins containing MCIDs likely form condensates by the cooperative effects of energetically favourable multivalent interaction of many copies of protein–protein interaction domains. These proteins are largely structured on the scale of amino acids; they have the stable tertiary structure of protein domains. They tend to be unstructured on the scale of the whole protein owing to flexible linkers between interaction domains [4,5]. In essence, they are a polymer made up of protein domain monomers. The interaction domains can undergo energetically favourable protein–protein interactions to drive phase separation [7]. Protein domains canonically interact through protein surfaces with complementary charged, hydrogen bonding and hydrophobic patches. Binding energies may reach 100 kJ mol^−1^ (near covalent bond strength, as for some antibodies), although may be much weaker. Interactions occur on spatial scales of globular proteins, around 1–10 nm. A single pair of interacting protein domains, equivalent to two monomers, would likely bind to each other and not phase separate. Proteins with multiple copies (i.e. MCIDs) can act like a polymer and undergo multivalent interaction to drive phase separation [4,5]. Synthetic MCIDs have reconstituted this behaviour; a mixture of polymerised small ubiquitin-like modifier proteins (polySUMO) and polySUMO-interacting motifs (SIMs) proteins generate condensates [36]. To date, condensates formed by MCID proteins have required two proteins with complementary interaction domains. This is somewhat analogous to a coacervate formed by two LCD scaffolds with complementary charge.

While seemingly being very different, MCID and LCD-containing proteins form condensates by analogous behaviours. Protein–protein interaction may occur on the amino acid or protein domain scale, with multivalency through repetitive sequences (LCDs) or multiple protein domains (MCIDs) being the defining hallmark of scaffolds that drive phase transitions. Either interaction mode can provide an energetic benefit to overcome the entropic cost of demixing. Recent studies have established that even in linear multivalent proteins, the synergy between interaction domains and intrinsically disordered linkers is crucial for determining whether these systems form gels driven by phase separation or if they form gels without undergoing phase separation [37]. In effect, the linkers generate multivalency and their sequence-encoded interactions control the degree and nature of cooperativity of interactions among protein interaction domains, thus influencing the nature of the phase transitions and the material properties of condensates.

## 4. Control of condensate composition

**Figure 1.**
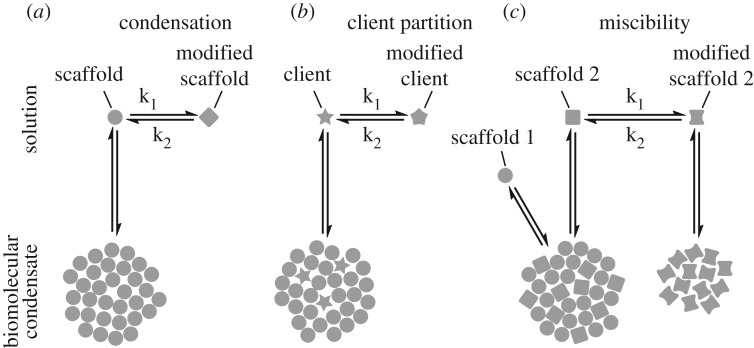
Equilibria underlying control of condensation, solvation and miscibility. Simplified models illustrating mechanisms controlling condensate dynamics and composition. (*a*) A scaffold protein is present at a low concentration in solution in dynamic equilibrium with the condensate. Conversion to a modified scaffold (increased k_1_) that cannot form a condensate will change the equilibrium, causing condensate dissolution. The reverse (increased k_2_) will cause condensation. (*b*) Control of partition of a molecule into a condensate can be viewed as control of solvation of the molecule by the scaffold in the condensate. A solute is present in dynamic equilibrium between the surrounding solution and the condensate, at a concentration ratio dependent on the partition coefficient. Conversion of the solute to a modified form that is insoluble in the condensate (increased k_1_) will cause partition away from the condensate. The reverse (increased k_2_) causes partition to the condensate. (*c*) One way in which multiple classes of condensates may be controlled through miscibility/immiscibility. A condensate made up of two miscible scaffolds exists in dynamic equilibrium with the low concentration of each scaffold in the surrounding solution. Conversion of scaffold 2 to a modified scaffold 2 (increased k_1_) will cause partition of scaffold 2 from the droplet (as in *a*). The modified scaffold 2 may be able to form a second condensate immiscible with the first, and increased k_1_ would promote this. The reverse would occur on increased k_2_.

A cell must be able to control the composition of a condensate to confer its necessary biochemical functions. A condensate has the normal properties of a liquid (box 1). A model of partition of molecules into a condensate is therefore the relative solubility of that molecule in the condensate and the surrounding cytoplasm (or nucleoplasm, etc.). Control of partition therefore requires control of relative solubility (figure 1*b* and table 1). The *solute* is the molecule being partitioned (these have also previously been referred to as clients [36]). The two solvents are the condensate and the surrounding cytoplasm. In this system the scaffold protein(s) and their hydration shell define the solvent properties of the condensate. The cytoplasmic proteins define the solvent properties of the cytoplasm. Control of condensate composition is therefore fundamentally unlike the use of porins and transporters in a membrane-bound organelle.

Box 1. **Emergent properties of condensates.**

Liquid condensates have all the surface and bulk properties typical of liquids. Surface tension means that the droplet relaxes to spherical shape after perturbation. They have the ability to fuse to form larger droplets and drip (undergo fission) under external forces. The condensate has a viscosity that, combined with surface tension, defines shear and relaxation rates [2]. Condensates can wet surfaces [2], with the contact angle depending on the molecular interaction [38]. In cells, condensates appear to wet membrane surfaces [2] and cytoskeletal fibres [39].

The boundary of a condensate is a physical barrier in that it is an interface between two liquids, but does not have a separate bounding object/material. The condensate, which is a dense phase, is in equilibrium with a dispersed phase and this phase equilibrium defines the phase boundary; the scaffold protein is enriched in the condensate and depleted in the surrounding solution, with constant exchange between the two [2]. The boundary marks a sharp change in the solvent environment.

Polymers making up a condensate may undergo cross-interaction on several different spatial and time scales and may tangle. Condensates may therefore have anomalous viscosity on different time and spacial scales. They may also have viscoelastic properties; the properties known for gels. A condensate may have predominantly liquid-like properties on cellular time scales of seconds to minutes. High condensate viscosity would give more gel or glass-like properties on cellular time scales. Condensate behaviour may also have a time dependence, with a characteristic transition time scale from an initial liquid-like state to a ‘hardened’ gel or glass-like state [3], perhaps owing to kinetic trapping in a non-equilibrium state.

The characteristic properties of a phase are only well defined for a bulk made up of many molecules on a characteristic time scale, but condensates are small liquid phases or ‘pseudo phases’ made up from large molecules. Smaller condensates may therefore approach the lower size limit where, on the time scales where the few molecules in the condensate rearrange, macroscopic properties like viscosity are no longer relevant. Conversely, variable stoichiometry protein complexes (for example polysomes) may be approaching the upper size limit where macroscopic liquid properties become relevant.

In a condensate formed from MCID scaffolds, solutes that undergo energetically favourable interactions with the structured protein domains of the scaffold may become enriched in the condensate. These interactions may be protein–protein, protein–nucleic acid or protein–small molecule, depending on the solute. For example SUMO preferentially partitions to a polySUMO/polySIM condensate [36]. RNA preferentially partitions to stress granule protein (FUS, etc.) condensates, owing to the RNA recognition motif (RRM) domain of stress granule proteins [17]. Hypothetically, a condensate that includes an enzyme domain would be expected to bind and concentrate its substrate and non-modifiable analogues. Control of partition could be achieved by modification of the solute to promote or prevent interaction with the scaffold binding domains, or modification of the solute binding domain on the scaffold (figure 1). For example, for condensates including proteins with SH2 domains (phosphotyrosine binding) [4,5], a tyrosine-containing peptide would be concentrated in the condensate only when phosphorylated. For condensates based on SUMO/SIM interaction, SUMOylation of a protein would lead to its concentration in the condensate [36]. Protein domain interactions can be highly specific, and relative solvation by this mechanism could be similarly specific.

In a condensate formed by LCD scaffolds, solutes that undergo energetically favourable interactions with the amino acids in the LCD may become enriched in the condensate. This applies to amino acids abnormally common in the LCD. For example, proteins rich in basic and aromatic amino acids (arginine and phenylalanine) preferentially partition to condensates formed from DDX4, which has an acidic and aromatic-rich LCD amino acid composition [40,41]. Control of partition could be achieved by modification of the general solute properties (charge, hydrophobicity, etc.) to alter interaction with the residues in the scaffold LCD. This could be achieved by post-translational modification in the case of protein solutes. For example, for a DDX4 condensate, where the DDX4 LCD is rich in acidic amino acids, introducing negative charge by serine phosphorylation would promote concentration in the condensate. These general properties are more closely analogous to partition in hydrophobic/hydrophilic solvents, and are likely less specific.

Partition of a solute between solvents could be controlled by enzymes acting on solutes in the cell (figure 1*b*). Such modifications would shift the equilibrium of solute partition, leading to altered condensate composition. Control of partition to a condensate is therefore fundamentally different to membrane-bound organelles. The molecule must be modified to alter partition, but the modifying enzyme could be positioned anywhere in the cell. This is unlike a membrane pore or transporter that does not modify the substrate and must be positioned in the membrane. However, a similar mechanism can act to partition a molecule to membrane-bound organelles. For example, glucose is phosphorylated following import to the cytoplasm to prevent its export by glucose transporters. Conceptually, control of solute partition to a condensate could rapidly integrate cellular events, with the composition of a condensate controlled by many solutes undergoing different modifications at many different localizations through the cell.

## 5. Managing multiple condensate compartments

A cell may contain many different condensates with different functions, therefore the cell must be able to control whether these compartments can mix or merge. Control of whether two condensates can mix is a question of whether they are miscible or immiscible. This is equivalent to asking whether two scaffolds will contribute to forming one condensate or two upon phase separation (figure 1*c*). Whether two scaffolds will form one mixed condensate or two different condensates depends on the relative energetic benefit of cross- or self-interaction. Two small molecules form miscible liquids when the cross- and self-interactions they undergo are of a similar type and strength, making it energetically favourable for the two types of solvent molecule to mix. Dissimilar interactions lead to phase separation, with one or both molecules experiencing a large energetic benefit to interact with itself (see below). Control of formation of one or multiple condensates is therefore fundamentally unlike the control of formation of one or multiple membrane-bound compartments by membrane fission and fusion (table 1). The best characterized examples of immiscible condensates are the nested nucleolar condensates [27].

In liquids made of small molecules, miscibility and immiscibility can be predicted with some accuracy from molecule properties. For liquids made of small hydrophobic molecules, miscibility can be predicted from the strength of van der Waals interactions (the Hildebrand solubility parameter). Miscibility of small molecule polar liquids, which undergo van der Waals, polar and hydrogen bonding interactions, requires all three interaction types to be taken into account (the Hansen solubility parameter). Miscibility of liquid phases formed by polymers can also be predicted [42]. However, for polymers that have non-uniform properties and the capacity for many types of intermolecular interactions, a quantitative prediction becomes intractably complex. We argue that some qualitative predictions can likely be made.

Cells appear to maintain multiple immiscible condensates simultaneously, but this has not been rigorously demonstrated for most compartments. For example, stress granules and P-bodies coexist in the cytoplasm. Liquid-like chromatin and nucleolar compartments coexist in the nucleus [1,20–23]. In both cases, their behaviour under induced merging has not been tested. Furthermore, these compartments could be under active maintenance in a non-equilibrium state. Condensates can conceptually form nested compartments, so long as a condensate is immiscible with the surrounding condensate. Cells appear to use this. For example, the fibrillar centre, dense fibrillary component and granular component of the nucleolus form nested condensate compartments [27]. Predicting miscibility, like predicting solvation, depends only on the types of intermolecular interaction in the liquid. The interactions that drive condensate formation therefore define both partition of solutes and whether another condensate will be miscible or immiscible, although quantitative prediction of either is extremely complex in the complex cell environment. Control of condensate miscibility could therefore be achieved through similar post-translational modifications to control solute partition.

## 6. Control of condensate dynamics

**Figure 2.**
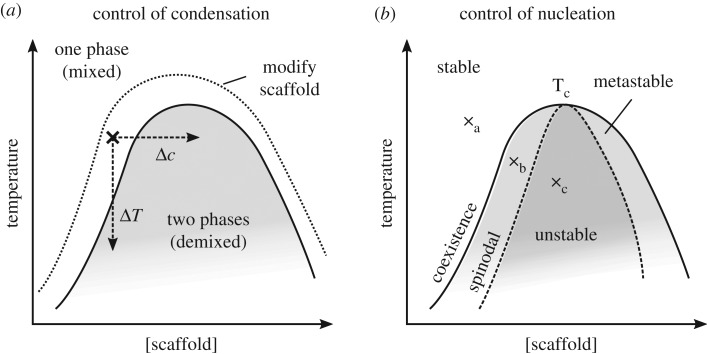
Methods to control condensation of a condensate. (*a*) Three example mechanisms for causing condensation for a cell with starting temperature and scaffold concentration indicated with a cross. The temperature shift Δ*T* would change the conditions to cross the phase boundary to a region allowing phase separation. Similarly, scaffold concentration change Δ*c* would allow phase separation. Alternatively, modification of the scaffold to change the phase diagram and move the phase boundary such that the current conditions cause demixing would also allow condensate condensation. (*b*) Under some conditions a demixed state is stable, but condensates do not spontaneously form. At point a the one phase mixed state is stable. At point b, across the phase boundary/coexistence curve, the one phase mixed state is metastable. Given a nucleator, demixing will occur. At point c, across the spinodal curve, the one phase mixed state is unstable and condensates will spontaneously nucleate.

Many cellular factors could alter the formation of condensates. Liquid condensates are dynamic and rapidly exchange proteins with the surrounding cytoplasm on timescales that are likely similar to or only few orders of magnitude slower than molecular processes such as folding and binding. Diffusion-limited exchange is possible over the entire surface. Small changes in the cytoplasmic environment could therefore have rapid effects on condensate formation and dissolution. Induction of condensate formation requires that the cytoplasm change to conditions where phase separation can occur, or that the scaffold proteins are modified such that the cytoplasm is an environment where condensation can occur (figures 1*a* and 2*a*). Phase separation is reversible, so reversal of the change that triggered condensation will drive dissolution. For example, in *Caenorhabditis elegans* embryos, micron-scale P granules can form/dissolve on the time scale of seconds to minutes [2]. This affords control of composition and enables integration of events from across the cell as modification of the scaffold could occur anywhere in the cell. Diffusion conveys this local change in ‘molecular identity’ of the scaffold to all regions of the cell to cause condensate dissolution or condensation. Suitable modifications to the scaffold, or changes to the cytoplasmic environment, to trigger phase separation can be predicted based on those that promote energetically favourable scaffold interactions.

For a condensate formed from a LCD scaffold, post-translational modifications that alter the properties of the LCD amino acids should modulate condensation. Large changes to chemical properties, rather than changes to residues' size or shape, are most likely to have an effect. For example, phosphorylation of serine residues alters serine from a polar to a strongly negatively charged phosphoserine. Hypothetically, serine phosphorylation may introduce favourable charge–charge interactions for a serine/arginine-rich LCD, or introduce unfavourable charge–charge repulsion preventing hydrogen bonding and hydrophobic interactions in a serine/tyrosine-rich LCD. Similarly arginine methylation may promote arginine–tryptophan interactions in an arginine–tryptophan-rich LCD [43].

For condensates formed from MCID scaffolds, post-translational modifications that introduce or remove binding sites should modulate condensation. This may be either by modification of amino acids or by addition of large adapters like ubiquitin or SUMO. For example, PML body formation requires SUMOylation of PML [44], and SUMO-2 and 3 include SUMOylation sites allowing polymerization of SUMO [45]. So-called polySUMO molecules, which are linear polymers comprising multiple SUMO domains connected by flexible linkers, can undergo multivalent interactions with polySIM and form condensates through networks of interactions among multiple polySUMO and polySIM molecules. Post-translational modifications that drive condensation, or reversal to drive condensate dissolution, could conceptually occur anywhere in the cell, thus providing dynamic control over the equilibrium between the condensates and the surrounding solution.

Changes to the chemical or physical properties of the cytoplasm may also trigger condensate formation or dissolution. This is equivalent to changing the position in the phase diagram from a region where phase separation will not occur to one where it will, or *vice versa* (figure 2). This may involve changes in scaffold protein concentration, temperature, concentrations of molecular crowders (equivalent to cell volume change or osmotic shock), or pH. For example, the concentration of cytoplasmic FUS increases in response to cellular stress, following export from the nucleus, and it condenses into stress granules [3]. Mechanical pressure on *Drosophila* embryos causes condensation of nuclear bodies, which may be triggered by crowding changes [46]. Hypothetically, condensates formed by hydrophobic interactions could act as a direct temperature sensor by dissolving at low temperatures. These condensates are likely more stable at high temperatures and less stable at low temperatures, owing to the entropic effects underlying hydrophobic interactions that become stronger at higher temperatures. This is similar to cold denaturation of globular proteins [47], but affecting intermolecular rather than intramolecular interaction. Methods for triggering condensation can also be synthetically engineered: a synthetic light-sensitive fusion protein, Cry2 fused with an LCD, forms condensates under illumination [48]. Direct sensitivity of condensates to the intracellular environment are therefore likely to be a new class of mechanisms in which cells can perceive their internal and external environment.

Condensation and dissolution will be sensitive to the concentration and properties of all other proteins and other biomolecules in the cell. This is an extremely complex contribution, as any molecule that readily interacts with the scaffold protein will alter the propensity to phase separate, and those that cannot will still contribute to molecular crowding. Molecules that undergo interactions that favour partition to a condensate will tend to affect the condensate more strongly [49]. In general terms, molecules that undergo low-valency interaction with scaffold proteins are likely to destabilize the condensate, while those that undergo high-valency solvation interactions would stabilize the condensate. This has been demonstrated in detail for poly-SUMO/poly-SIM condensates [36].

Condensation and dissolution, particularly for LCD scaffolds, should also be sensitive to small molecules in the cell. This is an emerging field, and limited evidence currently exists. However, the nature of the energetically favourable scaffold protein interactions that drive phase separation suggests some predictions. For condensates formed owing to favourable hydrophobic interactions, surfactants and hydrotropes may promote phase separation at low concentration (by helping solubilize the condensate in the surrounding hydrophilic cytoplasm) and prevent it at high concentrations (by solubilizing individual proteins forming the condensate). Many biological surfactants (bile acids, surfactants) exist and ATP is a potent hydrotrope [50]. This implies that cells have significant capacity to modulate condensates using these molecules, and there may be intrinsic links with energy availability in the cell [50]. For condensates formed by favourable charge–charge interactions, monovalent ions and small molecules would reduce multivalent charge–charge interaction, preventing condensate formation. Multivalent ions may promote condensation by coacervation or formation of salt bridges. For example, spermine and spermidine are polycationic natural metabolites abundant in the nucleus, which appear to interact with nucleic acids to allow phase separation [9]. Conversely, for condensation prevented by charge–charge interaction, monovalent ions may shield charge–charge repulsion to promote condensation. For condensates formed by polar or hydrogen bonding interactions, chaotropes, which reduce the effective strength of hydrogen bonding, would prevent condensate formation. Chaotropic properties are common in metabolites, including urea and alcohols. Therefore, there seems to be extensive capacity for a cell to modulate droplet formation with metabolites. It may be possible to exploit this for artificial modulation of condensate formation with synthetic small molecules; i.e. drugs.

Larger-scale interactions driving condensation may also be strengthened or weakened by small molecules. LCD scaffolds may form secondary structure that contributes to condensate formation [16]. Molecules that stabilize or destabilize this structure would be expected to modulate condensate formation. Trifluoroethanol stabilizes coiled coil interactions [51]. Therefore, it may promote condensates formed owing to energetically favourable transient coiled coil interactions, like TDP43 [16]. Conversely, molecules including anthraquinones and aminoacridines disrupt beta sheets in prion fibrils [52–54]. These may dissolve FUS condensates if transient beta sheet interactions contribute to condensation. For condensates formed from MCID scaffolds, molecules that cause canonical allosteric or orthosteric prevention or stabilization of domain–domain interaction would be expected to modulate condensation. For example, SUMO/SIM-binding small molecules in development [55] would drive droplet dissolution. Again, it may be possible to exploit this for artificial modulation of condensates by small molecules.

## 7. Control of condensate nucleation

Phase separation is greatly accelerated by nucleation. Even when phase separation is thermodynamically favourable, spontaneous (homogeneous) nucleation is extremely unlikely and the system can persist in a metastable well mixed, albeit supersaturated state (figure 2*b*). Routes to nucleate phase separation provide an additional point of control for condensate formation. Some cellular components that may promote nucleation can be predicted from the intermolecular interactions driving condensation.

Classical nucleation theory describes nucleation as overcoming the energy barrier of a liquid droplet reaching a sufficient surface area and surface energy, and heterogenous nucleation on a surface reduces this energy barrier [38]. Surfaces onto which a liquid wets more effectively, giving a smaller contact angle, reduce the volume (and number of liquid molecules) necessary to reach this surface area. A strongly wettable surface has surface properties that undergo favourable interactions with the liquid droplet. Transferring this concept to biomolecular condensates allows prediction of which cellular surfaces could nucleate particular condensates. For example, negatively charged structures (nucleic acid, membrane) should interact favourably with the basic C terminal LCD of *C. elegans* RNA binding protein PGL-3. This matches the observed behaviour of condensate nucleation on the nuclear envelope [2]. As another example, the MEG proteins appear to be necessary to nucleate liquid drops of PGL proteins [56] and the centriole is necessary to nucleate condensates of the centrosome protein SPD-5 [14]. Nucleic acids and analogues (poly-ADP ribose) should interact favourably with the low-specificity nucleic acid binding RRM domain of FUS. This matches the observed behaviour; poly-ADP ribose mediates FUS condensation at sites of DNA damage [3]. For proteins, interaction with the surface does not need to be the same as the ones driving condensation. For example, binding of the SH3 domain of Nck to phosphotyrosine residues in membrane-anchored p-Nephrin allows favourable interaction with a p-Nephrin coated bilayer. This provides a favourable surface on which Nck/N-WASP condensates can nucleate [6]. Modulation of these nucleation points by a cell could provide an additional point of regulation of condensation.

## 8. Conclusion

Recognizing that non-membrane-bound liquid intracellular compartments are liquid condensates and understanding the intermolecular interactions that drive their formation is highly informative. From the types of interaction, we suggest it is becoming possible to make specific predictions: the post-translational modifications that will promote condensation or dissolution, which molecules would preferentially partition to a condensate, and how those molecules may nucleate, stabilize or destabilize a droplet depending on their properties. We have focused on liquid condensates, but liquids formed by polymer phase separation often have viscoelastic properties and may be gel or glass-like on different spatial or time scales. This adds more complexity, although we argue the same principles often apply.

The emergent properties of condensates give them the capacity to compartmentalize biochemistry and achieve this through spatial and temporal control. In many cases, condensation is completely reversible, making the control of condensation or dissolution potentially extremely dynamic. Unlike membrane-bound organelles, condensates may be directly sensitive to changes in the cellular environment, post-translational modification, concentrations of small metabolites, interaction with other proteins and polymers etc. This makes the concepts for control of the dynamics of a condensate completely unlike membrane-bound organelles (table 1): condensates are sensitive to the cytoplasm environment, while membrane-bound organelles are stable under nearly all physiological conditions. Modification or generation of a molecule to promote or prevent condensation could act anywhere in the cell, and the change to the equilibrium will create or destroy condensates, while membrane-bound organelles must be directly created or destroyed. Finally, destruction (dissolution) of a condensate can be rapidly reversed, while destruction of a membrane-bound organelle cannot.

The dynamics of condensate formation, compositional control and control of miscibility make them an ideal rapid cellular response integrating information from multiple regions of the cell. Post-translational modifications of the scaffold and solute/client molecules anywhere in the cell can be rapidly transmitted through the cell by diffusion, in turn controlling the condensation, dissolution and composition of the corresponding condensates in all accessible areas of the cell.

Small molecules that alter condensate formation are beginning to be identified [50], and candidate molecules can be predicted from the interactions that generate the condensate. It is likely that cells use small molecules to control condensation and to modulate their properties, and it is plausible that synthetic small molecules could be designed to allow artificial control over condensate properties. Many condensates are associated with management of RNA and stress response, and several are made up of proteins associated with neurodegenerative disease [57,58]. We propose that further understanding the intermolecular interactions that drive condensate formation will allow us to understand pathogenesis and design interventions for these diseases.

## Data accessibility

This article has no additional data.

## Glossary

Biomolecular condensate *or* condensate: A non-membrane-bound compartment formed by phase separation
Scaffold: A protein or small group of proteins necessary for phase separation
Solute *or* client: A molecule that may or may not preferentially partition to a condensate, depending on its properties
